# The effect of music therapy on treating patients pain and anxiety in emergency department: a randomized controlled trial

**DOI:** 10.1186/s12245-025-00878-4

**Published:** 2025-04-11

**Authors:** Chuenruthai Angkoontassaneeyarat, Panatsaya Detsurang, Piraya Vichiensanth, Phanorn Chalermdamrichai, Arrug Wibulpolprasert, Natsinee Athinartrattanapong, Phatthranit Phattharapornjaroen, Natee Chiengchana, Wiputh Kehasuwan, Gritsada Huncharoen, Kanokkan Pothilert, Preedaporn Thipnangrong, Sirinat Loungnarin, Chaiyaporn Yuksen

**Affiliations:** 1 Department of Emergency Medicine, Faculty of Medicine, Ramathibodi Hospital, Mahidol University, Bangkok, 10400 Thailand; 2 College of Music, Mahidol University, Nakhon Pathom, Thailand; 3 Nursing Department, Faculty of Medicine, Ramathibodi Hospital, Ramathibodi Hospital, Mahidol University, Bangkok, 10400 Thailand

**Keywords:** Music therapy, Pain management, Anxiety reduction, Emergency department

## Abstract

**Background:**

Music therapy (MT) is a recognized modality for pain and anxiety reduction. Although its efficacy has been demonstrated in various clinical settings, its application in emergency departments (ED) remains controversial. This study aims to study the effects of MT in reducing pain and anxiety among patients visiting the ED with pain complaints.

**Methods:**

A single-center, randomized controlled trial was conducted at Ramathibodi Hospital, Bangkok, from July 2023 to September 2024. During each month of the study period, three days were randomly selected for the MT group, and another three days were designated for the non-MT group. All participants received standard analgesia and completed pre- and post-session questionnaires to assess pain, anxiety, satisfaction, and ED service quality before and one hour after analgesia. The MT group received MT sessions, each lasting 30–40 min.

**Results:**

Sixty-three patients participated (31 MT group, 32 control group). The MT group showed a significant reduction in pain scores of 1.52 points compared to 0.09 in the non-MT group (p 0.002). Anxiety score was also significantly reduced in the MT group by 1.87 points compared to 0.44 points in the non-MT group (p 0.026). The most significant improvements were observed in non-trauma-related pain and anxiety. Satisfaction scores increased in both groups (0.48 vs. 0.47 points; p 0.946), with no significant difference. However, MT significantly improved perceived ED service quality (0.98 vs. 0.10 points; p 0.001).

**Conclusion:**

In this study, we found that music therapy, when combined with standard analgesia, effectively reduced pain and anxiety in patients presenting to the ED, particularly those with non-trauma-related pain.

**Clinical trial number:**

TCTR20231109003. Registration site Thai Clinical Trials Registry. URL: https://www.thaiclinicaltrials.org/show/TCTR20231109003. Date of approval: 20 June 2023.

**Supplementary Information:**

The online version contains supplementary material available at 10.1186/s12245-025-00878-4.

## Introduction

Music therapy (MT) is a recognized modality for pain reduction. It utilizes the structured application of melody, rhythm, pitch, and dynamics as a supportive intervention. MT can be integrated with standard treatments, used during surgical procedures [1, 2], combined with pharmacological approaches, or employed as a primary alternative to conventional analgesics by providing distraction [3, 4]. Regarding the functions of music on pain, various studies have demonstrated that music significantly reduces the perceived intensity of pain, providing considerable relief for patients [3, 4]. A key mechanism underlying this effect is music’s ability to stimulate the secretion of endorphins and other “happy hormones.” Beyond its direct physiological effects, music serves as a distraction, helping individuals shift their focus away from pain sensations. Additionally, music promotes relaxation, leading to a reduction in heart rate and blood pressure, while also acting as an emotional regulator to manage stress and anxiety, both of which can exacerbate pain [5, 6].

Previous research has shown that MT effectively reduces pain. In 2018, a meta-analysis of 92 randomized controlled trials demonstrated that MT significantly decreased anxiety and pain during invasive surgery compared to control groups [7]. However, another randomized controlled trials that examined the effect of intra-operative stress reduction by MT in patients undergoing varicose vein surgery found no significant differences in heart rate gradient and blood pressure measured before and after the operation [8]. Additionally, a study involving end-stage palliative care patients used live music-based relaxation exercises in the intervention group and verbal relaxation exercises in the control group [9]. This study found that the levels of relaxation and well-being scores were significantly higher in the intervention group, but there were no differences in pain scores or heart rate variability.

Additionally, there were still some controversies in the utilization of MT in the emergency department (ED). One study in 2019 found that MT could reduce pain and stress and improve satisfaction, as measured by the Press Ganey ED survey, in over 1,500 patients who visited the ED in the United States, and 80% of patients stated that music therapy improved their caregiving experiences [10].

Among patients visiting the ED, 40–75% present with pain-related complaints [11–13]. Ramathibodi Hospital, a super tertiary care facility affiliated with Mahidol University in Bangkok, Thailand, handles approximately 40,000 annual ED visits and continues to face challenges in managing pain and stress effectively. No studies in Thailand have explored MT for ED patients, and existing studies have controversial results. Furthermore, cultural diversities in Thailand may also affect outcomes. The objectives of this study aim to study the effects of music therapy in reducing pain among patients visiting the ED at Ramathibodi Hospital by comparing pre-session and post-session pain scores. The secondary objectives were to study the effects of music therapy in reducing anxiety and increasing satisfaction and ED quality scores by comparing pre-session and post-session questionnaires on anxiety, satisfaction, and ED quality.

## Methods

### Study design and setting

This single-center, randomized controlled trial was conducted in the ED of Ramathibodi Hospital using pre-test and post-test questionnaires. Data were collected during the day shift (8:00 AM–4:00 PM) for the convenience of the music therapist. The study was conducted during official working hours, from Monday to Friday, spanning the period from July 1, 2023, to September 30, 2024.

Ramathibodi Hospital serves as a leading super-tertiary care institution, recognized for its pivotal role in medical education, training, and referral services. The hospital accommodates over 5,600 outpatient visits daily and houses more than 1,300 inpatient beds. The ED handles over 4,000 visits per month, with around 10% involving life-threatening conditions and 30% categorized as high-risk cases. Additionally, trauma cases account for approximately 10% of the total ED visits [14]. 

The Institutional Review Board of the Faculty of Medicine at Ramathibodi Hospital granted ethical approval for this experimental investigation, with the approval code COA MURA2023/449. Each participant provided written informed consent.

### Study participants

Adult patients presenting to the ED with pain during the study period were enrolled based on the following inclusion criteria: age ≥ 18 years, self-reported pain score of 1–6 at triage using the Numerical Rating Scale (NRS), ability to communicate verbally, stable vital signs as determined by the attending physician, completion of initial evaluation by ED providers, receipt of analgesics during the ED visit, and diagnosis of a non-surgical condition during the same visit.

Exclusion criteria included patients who declined to provide consent, those for whom MT could delay standard treatment, or individuals diagnosed with psychological or cognitive disorders that precluded participation in MT sessions.

### Randomization/ study protocol

#### Interventions

In the intervention (MT) group, participants received standard treatments from healthcare providers in the ED, including analgesia, before being transferred to a designated room in the ED. A pre-test questionnaire was administered to collect baseline data on pain scores, anxiety levels, satisfaction, and ED quality assessments (using a 0–10 scale). If participants were unable to write, the researchers assisted by reading the questions aloud, allowing for verbal responses. MT sessions, lasting 30–40 min, were conducted on a one-to-one basis by certified music therapists in a private treatment room within the ED. A post-test questionnaire was administered one hour after the administration of analgesia and completion of the MT session.

Throughout the study, music therapists maintained communication with ED physicians regarding any changes in participants’ clinical conditions. ED personnel had the authority to interrupt the session if necessary to ensure patient safety. If a medical emergency improved, the music therapist would consult with ED personnel before resuming the study protocol. In cases where ED personnel deemed that an eligible patient could no longer participate in the study after randomization, the patient was excluded post-randomization.

The control group also received standard treatment from healthcare providers in the ED and was given analgesia, then moved to the assigned room in the ED and provided the pre-test questionnaire. The research team asked for a post-test questionnaire 1 h after analgesia without the MT session.

The MT intervention in this study was conducted by a certified board-certified music therapist, who also served as one of the study investigators. A patient-centered approach was employed, customizing the therapeutic environment to address each participant’s individual needs and preferences. Participants actively engaged in the process by selecting music or activities that reflected their personal backgrounds, while the therapist cultivated a supportive and trusting relationship, empowering participants to take an active role in guiding their healing journey.

Before initiating the intervention, the music therapist introduced themselves, explained the objectives of the session, and provided an overview of the planned activities. Participants were asked about their song preferences, with the freedom to choose songs they enjoyed. The selected songs predominantly included various genres of Thai music, such as Luk Krung (urban music), Luk Thung (country music), and popular Thai hits. The core activities during the sessions involved music listening, singing, and reminiscing, with verbal interaction and emotional support encouraged between songs. All music was performed live by the therapist, who both sang and accompanied themselves on the guitar. At the conclusion of the session, participants were invited to reflect on their feelings and share their experiences from the therapy. (Supplement 1)

#### Randomization

A computer-generated random allocation sequence was used to determine study days each month. Three days per month were randomly assigned to the MT group, while another three days were designated for the non-MT group. On these predetermined study days, eligible patients were enrolled and assigned to either the MT or non-MT group accordingly. A maximum of two eligible patients could be enrolled per randomized study day. Each eligible patient, a detailed explanation of the study protocol was provided, and informed consent was obtained.

#### Blinding

Blinding was not implemented for eligible patients, physicians, or outcome assessors.

### Data gathering

Patients’ characteristics (age, gender, educational level, employment, pain score at triage, type of pain, visit time, time to analgesia, and type of analgesia) were documented using the questionnaire and electronic medical records. The outcome parameters (pre- and post-session pain scores, anxiety scores, patient satisfaction scores, and ED quality scores) were documented using the questionnaire.

We used a Thai-language questionnaire to collect patients’ characteristics and assess pre- and post-session scores. The questionnaire was reviewed and validated by a panel of ten experts, including emergency physicians and emergency medicine residents from the Department of Emergency Medicine, Ramathibodi Hospital.

Pain, anxiety, patient satisfaction, and ED quality scores were assessed using a 0–10 rating scale for each item. For pain, 0 represented “no pain,” and 10 represented “the most severe pain imaginable.” For anxiety, 0 indicated “no stress,” and 10 indicated “the highest stress possible.” For patient satisfaction, 0 indicated “not satisfied at all,” and 10 indicated “most satisfied.” For ED quality, 0 indicated “no quality at all,” and 10 indicated “the best quality possible.” Pre- and post-assessment forms were identical (Supplement 2).

### Outcome measurement

The primary outcome of this study was the difference in pain scores assessed before and after participants received analgesia in both the MT and control groups. The secondary outcomes included the differences in anxiety, satisfaction, and ED quality scores, assessed before and after participants received analgesia in both groups.

### Statistical analysis

#### Sample size Estimation

The sample size for this study was determined based on a previous study conducted by Parlar et al. [15], which evaluated the effect of MT on pain, anxiety, and patient satisfaction in patients presenting to the ED. In that study, the mean pain score in the MT group was 4.63 (SD 2.08), and the mean pain score in the control group was 6.00 (SD 1.74). For the sample size calculation, we compared two means in a non-inferiority or superiority framework using the formula below. We applied an alpha level of 0.05, a power of 0.8, and a sample size ratio of 1:1, resulting in a required total sample size of 31 participants per group.

#### Statistical analysis

The data were recorded using Microsoft Excel 2023, and statistical analyses were conducted using Stata version 16.0. We used a non-inferiority trial comparison to analyze the outcomes. Categorical variables are presented as counts and percentages (%), while continuous variables are expressed as either means with standard deviations (SD) or medians with interquartile ranges (IQR), depending on the distribution of the data. We tested hypotheses using Chi-Square and Fisher’s exact test for categorical data and paired t-test for continuous data, with a p-value < 0.05 for all analyses.

## Results

**Fig. 1:**
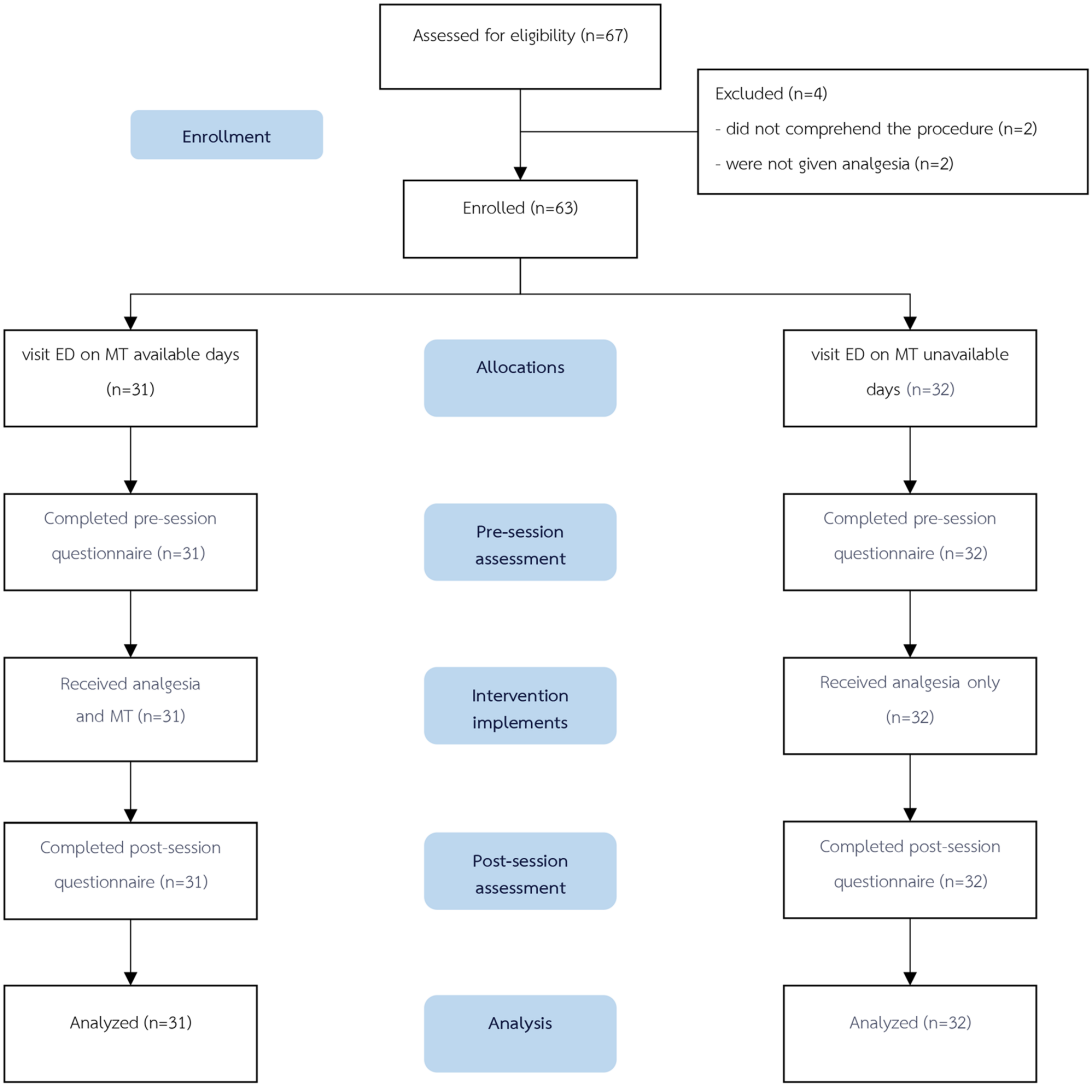
Study flow chart

Sixty-seven patients were assessed for eligibility. Four patients were excluded because they were not given analgesia (two patients) and did not comprehend the procedure (two patients). Therefore, the final trial consisted of 63 patients with a mean age of 58.25 ± 19.31 years, of which 38.46% were male. Thirty-one patients were assigned to the MT group, and the remaining 32 were assigned to the control group. (Fig. 1)

### Baseline characteristics of the study participants

The demographic and clinical characteristics of these patients are shown in Table 1. Participants in the MT group and the non-MT group were not significantly different between age (p 0.215), gender (p 0.541), level of education (p 0.684), occupation (p 0.544), type of pain (p 0.609), medication in each category; non-steroidal anti-inflammatory drugs (NSAIDs), weak opioids, opioids, and antispasmodic drugs (p 0.518, 0.585, 0.082, and 0.492, respectively). The most frequently used analgesic agents in our study were weak opioid groups, such as tramadol (71.43% overall, 67.74% in the MT group, and 75% in the non-MT group). Opioids (14.29% overall), including both oral and injectable forms of morphine, as well as NSAIDs (11.11% overall) like injectable and oral forms such as parecoxib or etoricoxib, were found to be used less frequently in this study.

**Table 1:** Patients baseline characteristics

Patient characteristic	Total (*n* = 63)	MT group (*n* = 31)	Non-MT group (*n* = 32)	*P*-value	
Age, (mean ± SD), years	58.25 ± 19.31	61.32 ± 18.79	55.25 ± 19.65	0.215	
Male, n (%)	25 (38.46)	12 (36.36)	13 (40.62)	0.541	
Educational level, n (%)					
Primary school and lower	16 (25.40)	8 (25.81)	8 (25.00)	0.684	
High school	14 (22.22)	8 (25.81)	6 (18.75)		
Diploma’s degrees	8 (12.70)	4 (12.90)	4 (12.50)		
Bachelor’s degrees	22 (34.92)	9 (29.03)	13 (40.62)		
Master’s degrees	3 (4.76)	2 (6.45)	1 (3.12)		
Occupation, n (%)					
Unoccupied	14 (22.22)	5 (16.13)	9 (28.12)	0.544	
Self-employed	12 (19.05)	7 (22.58)	5 (15.62)		
Merchant	8 (12.70)	5 (16.13)	3 (9.38)		
Civil servant	8 (12.70)	1 (3.23)	7 (21.88)		
Retired officer	12 (19.05)	10 (32.26)	2 (6.25)		
Business owner	7 (11.11)	2 (6.45)	5 (15.62)		
College student	2 (3.17)	1 (3.23)	1 (3.12)		
Type of pain*, n (%)					
Trauma-related pain^a^	25 (39.68)	11 (35.48)	14 (43.75)	0.609	
Non-trauma-related pain^b^	38 (60.32)	20 (64.52)	18 (56.25)		
Medication, n (%)					
NSAIDs	7 (11.11)	3 (9.68)	4 (12.50)	0.518	
Weak opioids	45 (71.43)	21 (67.74)	24 (75.00)	0.585	
Opioids	9 (14.29)	7 (22.58)	2 (6.25)	0.082	
Antispasmodic drugs	2 (3.17)	0 (0.00)	2 (6.25)	0.492	
Pain score at triage (mean ± SD)	5.39 ± 0.89	5.58 ± 0.62	5.22 ± 1.07	0.112	
Time in ED (median, range)	2.05 (0.55, 5.20)	2.00 (0.45, 4.58)	2.09 (0.55,5.25)	0.865	

**a**: Trauma-related pain, including fracture and accident-related musculoskeletal pain

**b**: Non-trauma-related pain, including abdominal pain, disease-related musculoskeletal pain, cancer pain, and pain from other causes

**Abbreviations**: MT, music therapy; ED, emergency department; NSAIDs, nonsteroidal anti-inflammatory drugs

Non-trauma-related pain (38, 60.32%), which includes abdominal pain, disease-related musculoskeletal pain, cancer pain, and pain from other causes, was more common among study participants than trauma-related pain (25, 39.68%), which includes fracture and accident-related musculoskeletal pain.

The overall median time in ED from triage to discharge was 2.05 h (IQR: 0.55–5.20); in the MT group, it was 2.00 h (IQR: 0.45–4.58) and 2.09 h (IQR: 0.55–5.25) in the control group that no significance between two groups (p 0.865). Furthermore, there was no difference in the initial mean pain score at triage in the MT group, which was 5.58 ± 0.62, and 5.22 ± 1.07 in the control group (p 0.112).

### Comparing the pre-and post-session outcomes between case and control groups

#### Pain score

In the MT group, the pre-session pain score was 5.58, and the post-session pain score was 4.06, showing a reduction of 1.52 points (95% CI, 0.86 to 2.17). In the non-MT group, the pre-session pain score was 5.22, and the post-session score was 5.13, reflecting a decrease of 0.09 points (95% CI, -0.55 to 0.74). There was a significant difference in pain score reduction between the two groups (p 0.002).

When dividing trauma-related pain from non-trauma-related pain, we discovered significant differences in pain-lowering among patients who presented with non-trauma-related pain (*p* < 0.001). In non-trauma-related pain, the pre-session pain score in the MT group was 5.50, and the post-session pain score was 3.30, showing a reduction of 2.20 points (95% CI, 1.37 to 3.03). Meanwhile, the pre-session pain score in the non-MT group was 5.39, and the post-session score was 5.61, with a decrease of 0.22 points (95% CI, -1.10 to 0.65). (Table 2)

**Table 2:** Comparison of pre-and post-sessions pain, anxiety, satisfaction and quality of ED service score in MT and non-MT groups

Outcomes	MT group (*n* = 31)			Non-MT group (*n* = 32)				*P*-value
	Pre-session	Post-session	Change score	Pre-session		Post-session	Change score	
Pain score (mean [95%CI])								
Overall (*n* = 63)	5.58 [5.06, 6.10]	4.06 [3.54, 4.59]	1.52 [0.86, 2.17]	5.22 [4.71, 5.73]		5.13 [4.62, 5.63]	0.09 [-0.55, 0.74]	0.002
Trauma-related-pain^a^ (*n* = 25)	5.73 [4.97, 6.49]	5.45 [4.70, 6.21]	0.27 [-0.58, 1.12]	5.00 [4.33, 5.67]		4.50 [3.83, 5.17]	0.50 [-0.25, 1.25]	0.695
Non-trauma-related-pain^b^ (*n* = 38)	5.50 [4.88, 6.11]	3.30 [2.68, 3.92]	2.20 [1.37, 3.03]	5.39 [4.74, 6.04]		5.61 [4.96, 6.26]	0.22 [-1.10, 0.65]	< 0.001
Anxiety score (mean [95%CI])								
Overall (*n* = 63)	6.16 [5.26, 7.06]	4.29 [3.39, 5.19]	1.87 [0.97, 2.77]	6.22 [5.33, 7.11]		5.78 [4.89, 6.67]	0.44 [-0.44, 1.32]	0.026
Trauma-related-pain^a^ (*n* = 25)	5.54 [4.02, 7.07]	4.55 [3.02, 6.07]	1.00 [0.56, 2.56]	6.35 [5.01, 7.71]		6.00 [4.65, 7.35]	0.36 [-1.03, 1.74]	0.547
Non-trauma-related-pain^b^ (*n* = 38)	6.50 [5.39, 7.61]	4.15 [3.04,5.23]	2.35 [1.29, 3.41]	6.11 [4.94, 7.28]		5.61 [4.44, 6.78]	0.50 [-0.62, 1.62]	0.019
Satisfaction score (mean [95%CI])								
Overall (*n* = 63)	8.65 [8.20, 9.08]	9.12 [8.69, 9.57]	0.48 [0.17, 0.80]	8.38 [7.94, 8.81]		8.84 [8.41, 9.28]	0.47 [0.16, 0.78]	0.946
Quality of ED service score (mean [95%CI])								
Overall (*n* = 63)	8.25 [7.81, 8.70]	9.23 [8.78, 9.67]	0.98 [0.59, 1.35]	9.03 [8.59, 9.47]		9.13 [8.69, 9.56]	0.10 [-0.28, 0.47]	0.001

**a**: Trauma-related pain, including fracture and accident-related musculoskeletal pain

**b**: Non-trauma-related pain, including abdominal pain, disease-related musculoskeletal pain, cancer pain, and pain from other causes

Abbreviations: MT, music therapy; ED, emergency department

#### Anxiety score

Pre-session anxiety in the MT group was 6.16, while post-session anxiety was 4.29, indicating a 1.87-point decrease (95% CI, 0.97 to 2.77). Pre-session anxiety in the non-MT group was 6.22, while post-session anxiety was 5.78, a 0.44-point decrease (95% CI, -0.44 to 1.32). The reduction of anxiety levels between the two groups varied significantly (p 0.026).

Similar to the reduction in pain, there were significant differences in the reduction of anxiety scores in patients presenting with non-trauma-related pain (*p* = 0.019). In the MT group, the pre-session anxiety score was 6.50, and the post-session anxiety score was 4.15, reflecting a reduction of 2.35 points (95% CI, 1.29 to 3.41). In the non-MT group, the pre-session anxiety score was 6.11, and the post-session score was 5.61, with a reduction of 0.50 points (95% CI, -0.62 to 1.62) (Table 2).

#### Satisfaction score

The two groups had no difference in satisfaction score rises (p 0.946). In the MT group, the pre-session satisfaction score was 8.65, and the post-session score was 9.12, reflecting an increase of 0.48 points (95% CI, 0.17 to 0.80). In the non-MT group, the pre-session satisfaction score was 8.38, and the post-session score was 8.84, reflecting an increase of 0.47 points (95% CI, 0.16 to 0.78).

#### Quality of ED service score

A statistically significant difference in the quality of ED service scores was observed between the two groups, with the MT group showing a more substantial increase (p 0.001). In the MT group, the pre-session quality of ED service score was 8.25, and the post-session score was 9.23, reflecting an increase of 0.98 points (95% CI, 0.59 to 1.35). In the non-MT group, the pre-session score was 9.03, and the post-session score was 9.13, reflecting an increase of 0.10 points (95% CI, -0.28 to 0.47).

## Discussion

In this prospective randomized control trial study, the effects of MT on patients presenting to the ED with pain demonstrated that integrating MT with conventional analgesia can significantly reduce pain and anxiety compared to traditional analgesia alone, particularly in non-trauma-related pain. Moreover, MT may enhance ED quality scores. Patient satisfaction was growing, even if the difference was not statistically significant.

Our results showed a statistically and clinically significant reduction in pain scores compared between the MT and non-MT groups (1.52 points vs. 0.93 points; p 0.002). According to a study conducted in an ED in the United States, a difference of approximately 1.39 points on the NRS for pain is clinically significant and relevant for patients [16]. Similar to the previous studies conducted in the ED [15, 17], which found that MT can significantly reduce pain, particularly in non-trauma-related patients [10, 18]. One recent systematic review discovered that the factors of interventions of more than 20 min of MT, as our interventions, were also associated with reduced pain scores [19]. Pain from trauma produced from local tissue damage stimulates somatic pain receptors that cause sharp pain. It is more likely that patients presented with trauma-related pain should be treated with analgesic agents or surgery [20, 21]. Our result found no significant differences in pain reduction in the trauma group, which the pathophysiology of the pain itself could explain. According to the study of Duroux et al. [22], MT did not diminish pain during wound closure in the ED. MT, which can effectively reduce pain scores, may also reduce the requirement for pain prescriptions in the ED. As a result, this improves patient comfort and general well-being by reducing the adverse effects of drugs, particularly opioids or weak opioids, like nausea, vomiting, and dizziness [23, 24].

Additionally, MT also had a significant effect on anxiety scores. The MT group’s self-report anxiety score decreased by 1.87 points, while the non-MT group’s decreased by 0.44 points, according to our results. Comparable to pain reduction, the effect of MT for anxiety reduction is significant in patients with non-trauma-related pain. Regardless, current studies of MT show debate about its impact on anxiety. MT decreased anxiety levels for non-trauma-related pain in ED [10, 25–28] and pediatric populations [29, 30] but not during the procedure [22]. However, MT is probable to diminish the anxiety level of patients, as explained by the study by Thoma et al., who found MT impacted anxiety due to significant differences in cortisol response after being assigned to music listening [31]. The diversity of the population groups caused the different perceptions of the patients toward music and stress. Anxiety reduction not only benefits patients but also helps reduce stress among relatives and healthcare providers in the ED.

Our results showed no significant differences in satisfaction scores between the groups. Since the pre-session scores were high at 8.65 and 8.38 in the MT and non-MT groups, it likely reflected the high level of confidence and satisfaction that patients perceive for the services provided by Ramathibodi Hospital, which is a university-affiliated tertiary care hospital. However, there was a significant difference in the quality-of-service scores, indicating that MT can enhance service quality and treatment in the ED.

## Limitation

Our study has several limitations to consider. First, the single-center design may not reflect the broader population, limiting the generalizability of our findings to other settings. The limited study period, with data collection restricted to day shifts, may have excluded the experiences of patients visiting the ED during night shifts, weekends, or holidays. Lack of participant randomization due to scheduling constraints of the MT service, provided by board-certified therapists from the College of Music, Mahidol University, may have introduced selection bias. The small sample size of 63 participants limits statistical power and the ability to detect smaller effect sizes or rare outcomes. Lack of blinding of participants and healthcare providers may have introduced bias in outcome reporting. The intervention was delivered by a specific MT provider, a board-certified music therapist, which may not be representative of the skills and experience of other therapists, potentially affecting the generalizability of the findings. Subjective outcome measures, such as self-reported pain, anxiety, satisfaction, and service quality, may have been influenced by individual biases and perceptions. The cultural specificity of music, with Thai music used in the therapy sessions, may limit the applicability of the findings to different cultural contexts. Confounding factors, such as the ED atmosphere, overcrowding, noise levels, and personnel behavior, may also have influenced the results. Furthermore, pre-ED analgesic or sedative use could have affected pain and anxiety levels, influencing study outcomes. The study does not differentiate between chronic vs. acute pain, which may involve different underlying mechanisms and responses to MT. Lastly, trauma-related vs. non-trauma-related pain involves distinct pain mechanisms, and addressing these separately would help to avoid confounding results.

Despite these limitations, our study highlights the potential benefits of music therapy in ED, though its application should be tailored to specific clinical settings and available resources. Further research should address these challenges to ensure more robust and widely applicable findings.

## Conclusion

MT has shown benefits in reducing pain and anxiety when integrated with conventional analgesia in patients who presented the ED with complaints of pain, especially for non-trauma-related pain. MT did not significantly differ in self-report satisfaction scores. However, there was a significant difference in the quality-of-service scores, indicating that MT can enhance service quality and treatment of pain in the ED.

## Electronic supplementary material

Below is the link to the electronic supplementary material.

Supplementary Material 1

Supplementary Material 2

## Data Availability

No datasets were generated or analysed during the current study.

## Declarations

### Using artificial inteligence chatbots

During the preparation of this work the author(s) used ChatGPT4.0 in order to check and correct grammatical error during the manuscript writing process. After using this tool/service, the author(s) reviewed and edited the content as needed and take(s) full responsibility for the content of the publication.
