# Supplementary material for: The effect of music therapy on treating patients pain and anxiety in emergency department: a randomized controlled trial
Source: Int J Emerg Med. 2025 Apr 11;18:77. doi: 10.1186/s12245-025-00878-4 (PMC11992888; doi:10.1186/s12245-025-00878-4)
Supplement: Supplementary file 1 — Supplementary Material 1 [file 12245_2025_878_MOESM1_ESM.docx]

**Supplement 1**

**Pictures of music therapy sessions**


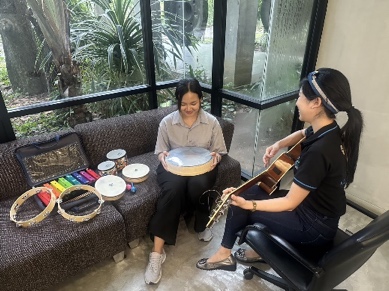

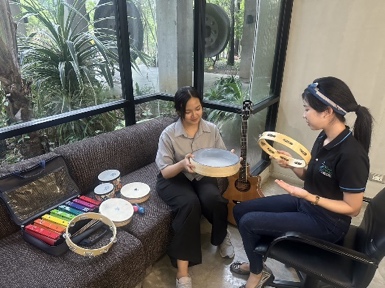

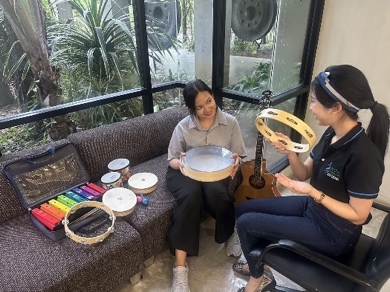
 A B C


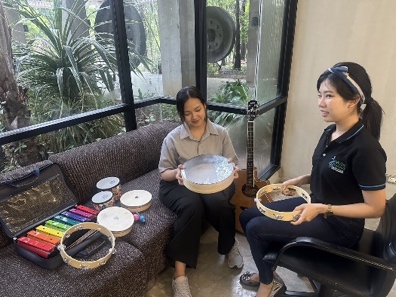

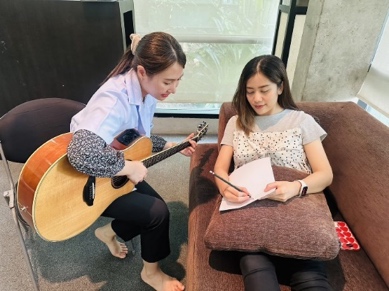

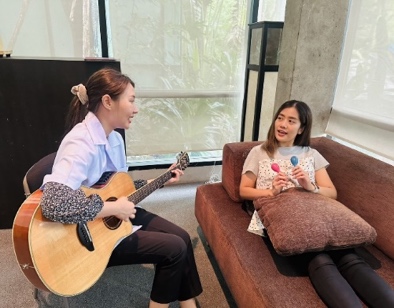
 D E F

G

**Figure A-E.** Music therapists play various instruments along with singing, and patients join in by singing or playing accompanying instruments.

**Figure F.** Patients also co-write songs with the music therapist.
