# Supplementary material for: The effect of music therapy on treating patients pain and anxiety in emergency department: a randomized controlled trial
Source: Int J Emerg Med. 2025 Apr 11;18:77. doi: 10.1186/s12245-025-00878-4 (PMC11992888; doi:10.1186/s12245-025-00878-4)
Supplement: Supplementary file 2 — Supplementary Material 2 [file 12245_2025_878_MOESM2_ESM.docx]

**Supplement 2**

**Patient record form 1.** Patient characteristics form

**
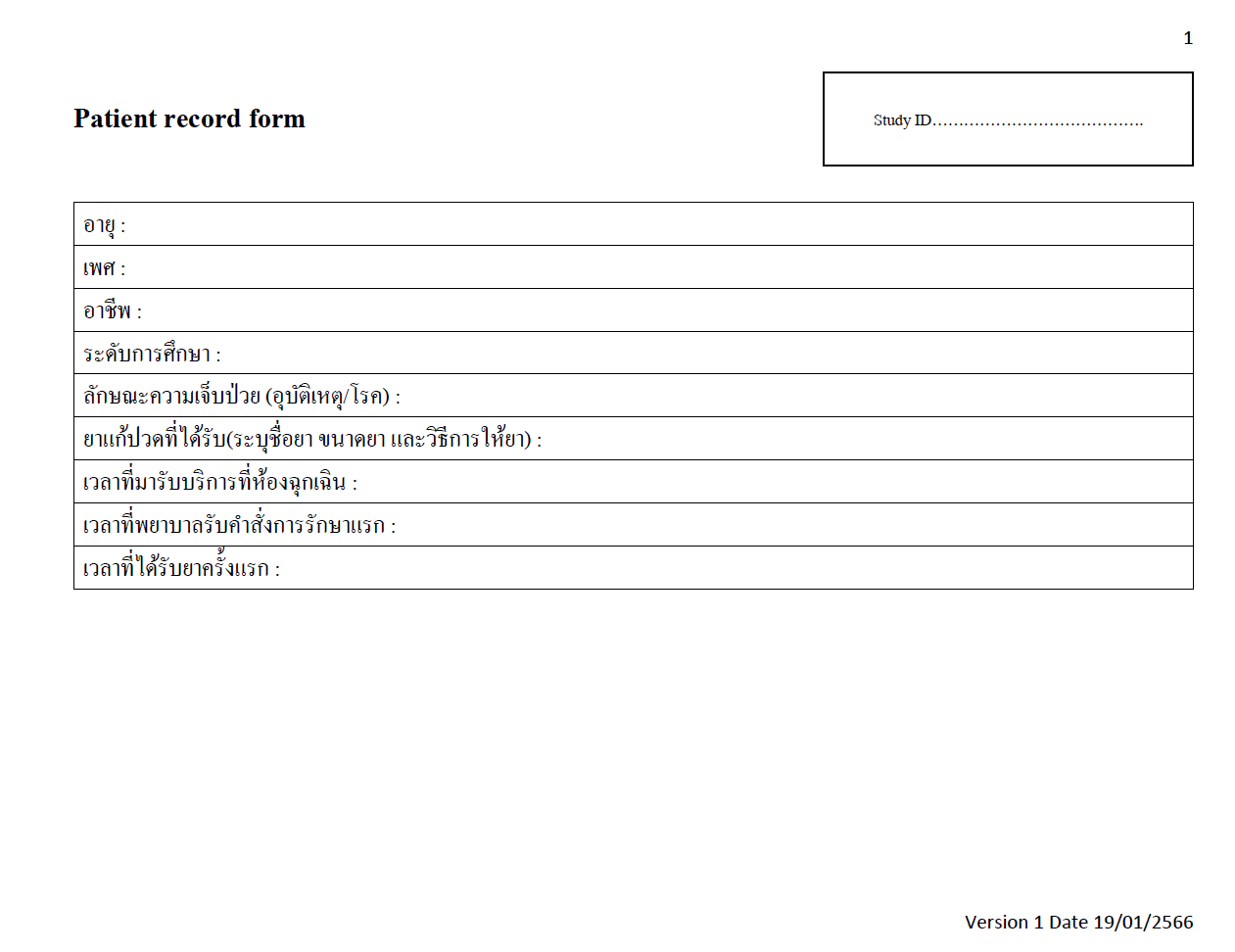
**

**Patient record form 2.** Music therapy data form


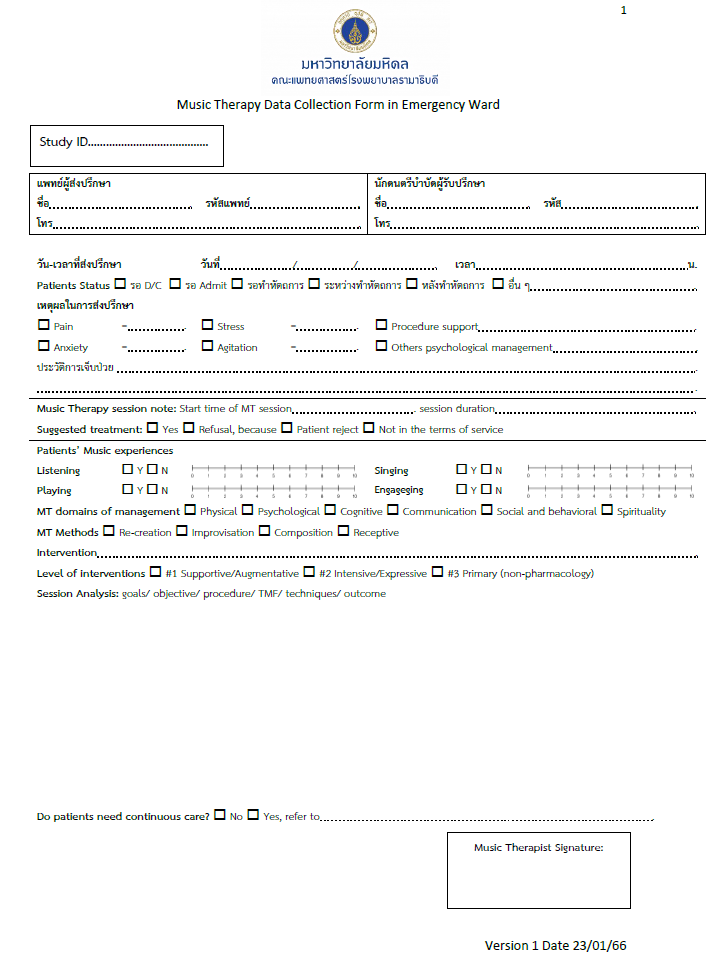


**Questionnaires 1.** Pre-session questionnaires form


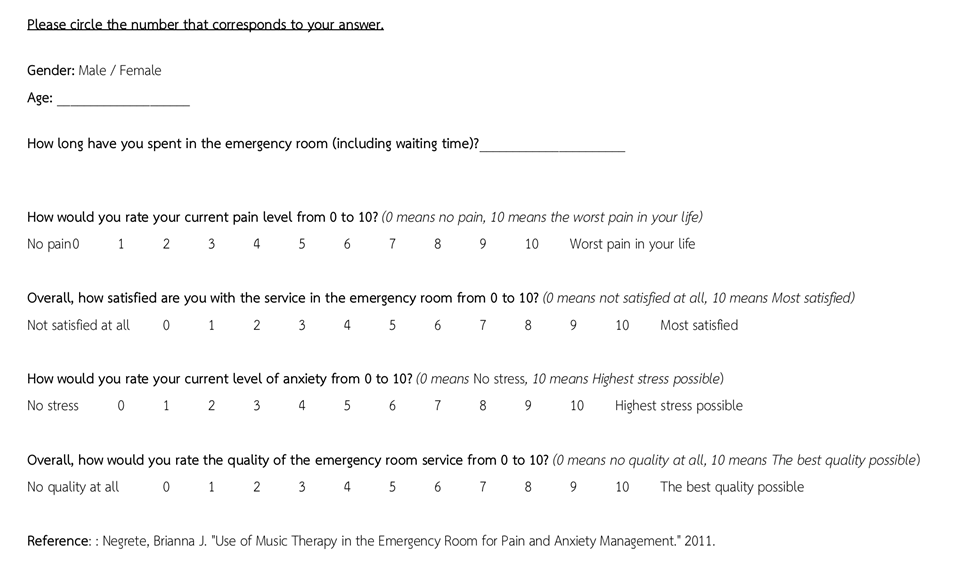


**Questionnaires 2.** Post-session questionnaires form

**
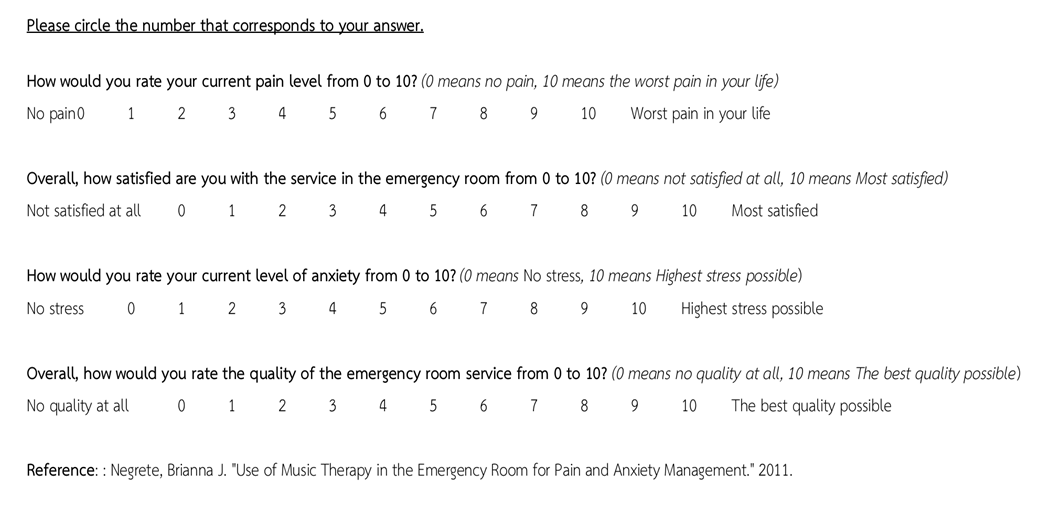
**
